# A highly selective pyridoxal-based chemosensor for the detection of Zn(ii) and application in live-cell imaging; X-ray crystallography of pyridoxal-TRIS Schiff-base Zn(ii) and Cu(ii) complexes†

**DOI:** 10.1039/d1ra05763d

**Published:** 2021-10-21

**Authors:** Anwar Hussain, Kadarkaraisamy Mariappan, Dawson C. Cork, Luke D. Lewandowski, Prem K. Shrestha, Samiksha Giri, Xuejun Wang, Andrew G. Sykes

**Affiliations:** Contribution from the Department of Chemistry, University of South Dakota Vermillion SD 57069 USA Andrew.Sykes@usd.edu; Basic Biomedical Science, University of South Dakota, School of Medicine Vermillion SD 57069 USA

## Abstract

In a simple, one-step reaction, we have synthesized a pyridoxal-based chemosensor by reacting tris(hydroxymethyl)aminomethane (TRIS) together with pyridoxal hydrochloride to yield a Schiff-base ligand that is highly selective for the detection of Zn(ii) ion. Both the ligand and the Zn(ii) complex have been characterized by ^1^H & ^13^C NMR, ESI-MS, CHN analyses, and X-ray crystallography. The optical properties of the synthesized ligand were investigated in an aqueous buffer solution and found to be highly selective and sensitive toward Zn(ii) ion through a fluorescence turn-on response. The competition studies reveal the response for zinc ion is unaffected by all alkali and alkaline earth metals; and suppressed by Cu(ii) ion. The ligand itself shows a weak fluorescence intensity (quantum yield, *Φ* = 0.04), and the addition of zinc ion enhanced the fluorescence intensity 12-fold (quantum yield, *Φ* = 0.48). The detection limit for zinc ion was 2.77 × 10^−8^ M, which is significantly lower than the WHO's guideline (76.5 μM). Addition of EDTA to a solution containing the ligand–Zn(ii) complex quenched the fluorescence, indicating the reversibility of Zn(ii) binding. Stoichiometric studies indicated the formation of a 2 : 1 L_2_Zn complex with a binding constant of 1.2 × 10^9^ M^−2^ (±25%). The crystal structure of the zinc complex shows the same hydrated L_2_Zn complex, with Zn(ii) ion binding with an octahedral coordination geometry. We also synthesized the copper(ii) complex of the ligand, and the crystal structure showed the formation of a 1 : 1 adduct, revealing 1-dimensional polymeric networks with octahedral coordinated Cu(ii). The ligand was employed as a sensor to detect zinc ion in HEK293 cell lines derived from human embryonic kidney cells grown in tissue culture which showed strong luminescence in the presence of Zn(ii). We believe that the outstanding turn-on response, sensitivity, selectivity, lower detection limit, and reversibility toward zinc ion will find further application in chemical and biological science.

## Abstract 2

The synthesis, characterization, X-ray crystallography, and live-cell imaging of pyridoxal-TRIS Schiff-base ligand which is selective as a luminescence sensor to detect Zn(ii) ion, and the corresponding Zn(ii) and Cu(ii) complexes are described.

## Graphical abstract

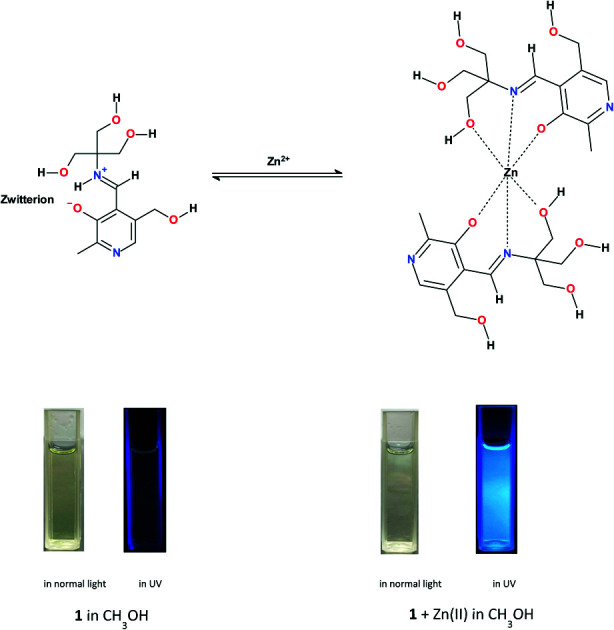

## Introduction

Zinc is the second most abundant metal in the human body, and plays a critical role in many biological processes such as gene expression, protein and DNA synthesis, enzymatic reactions, growth, and development processes, as well as in many pathological processes such as epilepsy, Alzheimer's disease, infantile diarrhea, and ischemic stroke.^1–6^ In addition, a high concentration of chelatable zinc ion is found in the brain, spermatozoa, and pancreas.^7–9^ Chelatable Zn(ii) has numerous benefits in biological systems such as regulating neuron transmission, suppressing apoptosis, immune function, *etc.*; however, the mechanism of chelatable Zn(ii) is less understood as compared to other cations such as Na(i), K(i), and Ca(ii). On the other hand, Zn(ii) is spectroscopically silent due to its closed-shell [Ar]3d^10^4s^0^ electronic configuration; hence detecting zinc with conventional techniques such as nuclear magnetic resonance (NMR), electron paramagnetic resonance (EPR), and electronic absorption spectroscopy is ineffective.^5^ Atomic absorption spectroscopy can detect zinc selectively and sensitively; however, this technique is destructive to the sample and has no spatial resolution.^10^ Analytical methods such as UV-Vis spectroscopy, potentiometry, and electrochemical analysis have also been used for the detection of Zn(ii), Cu(ii), and Co(ii).^11–13^ Fluorescent probes are the most effective tools for sensing applications, however, exhibiting high sensitivity and selectivity, and have generally short response times when imaging biological samples. Several fluorescent probes have been reported for the detection of zinc ion in a wide variety of cells such as A549 cells, SW620 cells, Vero cells, HeLa cells, MDA-MB 468 cells, HEK 293 cells, zebrafish, and several other cells with applications in bio-imaging.^14–22^ Additional studies have been carried out in similar alcohol/water mixtures as employed in the bio-imaging studies here.^20,23–25^

Dipicolylamine (DPA), coumarin, quinoline, bipyridine, triazole, iminodiacetic acid, acyclic and cyclic polyamines, and Schiff-bases are commonly used receptors to detect Zn(ii) ion.^5,26–30^ These receptors often contain nitrogen atoms which serve as good binding sites for Zn(ii), and when linked with various fluorophores, they can induce effective signaling transduction to recognize the binding event through a variety of photophysical mechanisms. In this regard, we have focused on an easily prepared Schiff-base ligand (one-step synthesis), using readily available reagents, that yields a fluorescent probe based on pyridoxaldehyde. Schiff-bases are generally poor/non-fluorescent due to conventional modes of non-radiative decay such as C=N bond isomerization and/or excited-state intramolecular proton transfer (ESIPT) involving phenolic protons.^31^ Pyridoxaldehyde is one form of vitamin B6 which acts as a co-enzyme in various enzyme reactions and is involved in aerobic metabolism.^32^ Pyridoxaldehyde also acts as a coenzyme in many enzymatic reactions and is involved in many aspects of macronutrient metabolism, neurotransmitter synthesis, histamine synthesis, hemoglobin synthesis and function, and gene expression. This suggests that pyridoxaldehyde is biocompatible and can be readily used in bio-applications. Several Schiff-bases based on pyridoxaldehyde have been reported in the literature that forms complexes with a wide variety of metal cations, including transition metals,^33–43^ heavy metals,^44,45^ lanthanides,^46–49^ and actinides.^49–51^ However, only a few reports show pyridoxal-based Schiff-bases act as fluorometric or colorimetric chemosensors for the detection of transition metals, biomolecules, and anions.^31,52–64^

**Scheme 1:**
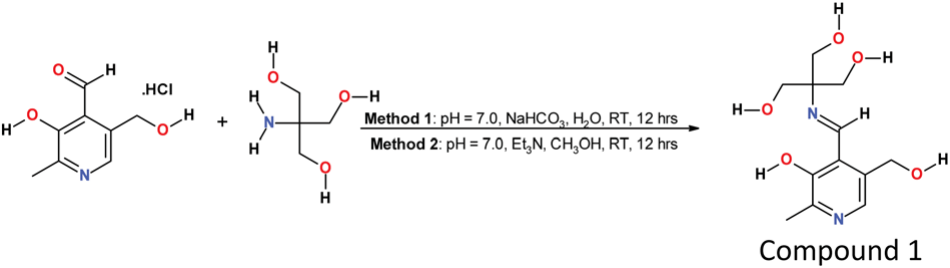
Synthesis of pyridoxal-TRIS Schiff's Base.

In this report, we explore a pyridoxal-based, Zn(ii) selective fluorescent probe combined with TRIS that exhibits, ease of synthesis, cost efficiency, chemical and photostability, fluorescence selectivity and sensitivity, and fast response time for spectral imaging of live-cells (Scheme 1). The coordination environment for Zn(ii) ion complexation is clearly delimited by stoichiometric studies and X-ray crystallography, and the TRIS functionality of the Schiff-base provides alternate hydroxyl binding sites for competing metal cations that are more oxophilic than Zn(ii), yielding exceptional selectivity and sensitivity.

## Materials and physical methods

Pyridoxal hydrochloride, 2-amino-2-(hydroxymethyl)-1,3-propanediol (TRIS), and triethylamine were purchased from Sigma-Aldrich and used without further purification. All other materials were of reagent grade. All cations used in this study are perchlorate salts, except tetrakis(acetonitrile)copper(i) tetrafluoroborate. Nuclear magnetic resonance (NMR) spectra were collected in DMSO-*d*_6_ at room temperature on a Bruker Ascend™ 400 MHz, and the chemical shifts were reported in part per million (ppm). Fourier transform-infra red spectroscopy (FT-IR) spectra were collected on a Bruker Alpha FT-IR spectrometer, and the wavenumber was reported in cm^−1^. Element analyses were carried out using an Exeter Analytic CE-440 and helium gas was used as the carrier gas. UV-Vis spectra were collected using a Varian Cary 50 Bio UV-Vis spectrometer and luminescence spectra were collected using a Photon Technology International (PTI) steady-state spectrofluorometer and Fluoromax-4. Binding constant determinations were conducted using Bindfit – Host/Guest modelling software freely available at supramolecular.org.^80^ Electrospray ionization-mass spectroscopy (ESI-MS) data were collected using a Varian 500-MS Ion Trap Mass spectrometer in dilute methanol. Crystallographic data were recorded using a Bruker Venture D8 Apex III diffractometer at 100 K using MoK_α_ radiation and CuK_α_. The crystallographic data were integrated by Apex III software, and the structures were solved using SIR97 (ref. 65) and refined with SHELXL-97 (ref. 66) followed by further refinement and completion of the structure on WinGX^67^ and Mercury 2020.3 Windows was used to visualize the structures. The alcohol group at C12 of 1 was modeled as disordered over two positions with 50 : 50 occupancies. Graphical figures were plotted using OriginPro 9.0 software. The reaction schemes and chemical structure were drawn on BIOVIA Draw for academic use by Dassault Systèmes.

### Synthesis of pyridoxal-TRIS Schiff-base ligand (1)

#### Method 1

0.5 g (2.45 mmol) of pyridoxal hydrochloride was dissolved in 25 mL of water, and the pH of the solution changed from 3 to 7 by the addition of sodium bicarbonate as monitored by pH indicator strips. 0.3 g (2.45 mmol) of TRIS is added and the reaction mixture was left overnight to stir at room temperature. After the completion of the reaction, the solvent was evaporated and the solid residue dissolved in methanol, and any undissolved solid residue is removed using gravity filtration. Excess diethyl ether is added to the methanol solution to obtain the product by precipitation, which is filtered and washed.

#### Method 2

0.2 g (0.98 mmol) of pyridoxal hydrochloride is dissolved in 10 mL of methanol, and the pH of the solution changed from 3 to 7 by adding approximately 20 drops of triethylamine as monitored by pH indicator strips. 0.12 g (0.98 mmol) of TRIS is added, and the reaction mixture was left overnight at room temperature. After the reaction was complete, excess diethyl ether was added and left in the refrigerator overnight to obtain the product as yellow crystals.

#### Compound 1

Percent yield: 84% (method 1) and 86% (method 2), melting points: 233–235 °C, molar mass: 270.28 g mol^−1^, elemental analyses for C_12_H_18_N_2_O_5_: theoretical C = 53.33%; H = 6.71%; N = 10.36% and experimental C = 53.17%; H = 6.69%; N = 10.42%, ^1^H NMR (400 MHz, DMSO-*d*_6_, 25 °C): *δ* 2.34 (3H, s, methyl), 3.62–3.64 (6H, dd, *J* = 5.20 Hz, methylene), 4.60–4.62 (2H, dd, *J* = 5.04 Hz, methylene), 4.88–4.91 (3H, t, *J* = 5.34 Hz, alkoxy), 5.30–5.33 (1H, t, *J* = 5.24 Hz, alkoxy), 7.72 (1H, s, aromatic), 8.89 (1H, s, carbimine), 15.44 (1H, s, zwitterion proton), ^13^C NMR (400 MHz, DMSO-*d*_6_, 25 °C): *δ* 162.48, 158.75, 150.76, 134.99, 133.07, 117.89, 68.02, 61.54, 58.96, 193.4, ESI-MS (CH_3_OH): 271.2 *m*/*z* [M + H]^+^.

### Synthesis of the Zn complex (2)

A methanolic solution of ligand (0.1 g, 0.37 mmol) was added dropwise to a solution of hydrated zinc perchlorate (Zn(ClO_4_)_2_·6H_2_O, 0.14 g, ∼0.37 mmol), and the mixture was stirred at room temperature. The resulting reaction was filtered, and the supernatant liquid was kept at room temperature for slow evaporation. A suitable pale-yellow single-crystal was obtained for X-ray structure determination. Percent yield: 35%.

### Synthesis of Cu complex (3)

A 5 mL methanolic solution of ligand (0.1 g, 0.37 mmol) was added dropwise to the 5 mL acetonitrile solution of hydrated copper perchlorate (Cu(ClO_4_)_2_·6H_2_O, 0.14 g, ∼0.37 mmol), and the mixture was refluxed for 4 h. Excess of diethyl ether was added to the reaction mixture and left in the refrigerator overnight. A green crystal was obtained, which is dissolved into acetonitrile and diffused into diethyl ether to get an X-ray quality crystal. Percent yield: 82%. Molar mass: 532.72 g mol^−1^, elemental analyses for C_12_H_18_N_2_O_13_Cl_2_Cu_1_: theoretical C = 27.06%; H = 3.41%; N = 5.26% and experimental C = 27.50%; H = 3.28%; N = 5.36%. ESI-MS (*m*/*z*) = 553.2 [M + Na]^+^ (cal. 553.93).

### UV-Vis and fluorescence studies

Both UV-Vis and fluorescence spectra were collected using the same solutions without altering the conditions. A stock solution of cation and anion salts were prepared in 10 mL (1.2 × 10^−2^ M) of acetonitrile or H_2_O, and a stock solution of compound 1 in 100 mL (5 × 10^−5^ M) in various solvent systems such as 100% methanol, 10% and 50% water (0.1 M HEPES buffer, pH = 7.3) in methanol were prepared. UV-Vis and fluorescence studies were executed by titration of 25 μL aliquots of stock cations or anions salt solution to 3.0 mL of 1 at room temperature. For the stoichiometric and binding constant study of Zn(ii) with 1, the stock solution of Zn(ii) is diluted to 6.0 × 10^−4^ M, and titration was carried by successive addition of 25 μL aliquots of Zn(ii) salt solution to 3.0 mL of 1.

### Quantum yield determination

We used anthracene in ethanol as the reference, and the following equation was used for the calculation of the quantum yield.^68,69^1
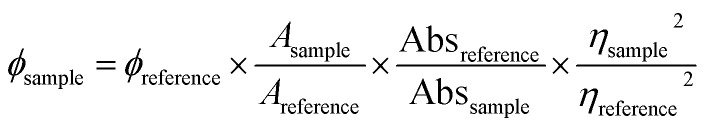
where, *ϕ*, Abs, *A*, and *η* represent quantum yield, absorbance, area under the fluorescence curve, and refractive index of the medium, respectively.

### Detection limit determination

The detection limit of 1 for Zn(ii) was determined by using fluorescence titration data at 472 nm based on a reported method.^70^ The detection limit was calculated based on eqn (2), where *σ* is the standard deviation of blank measurement, and *K* is the slope between the fluorescence intensity *versus* Zn(ii) concentration. The fluorescence spectrum of compound 1 was recorded 6 times to achieve the standard deviation of blank.2
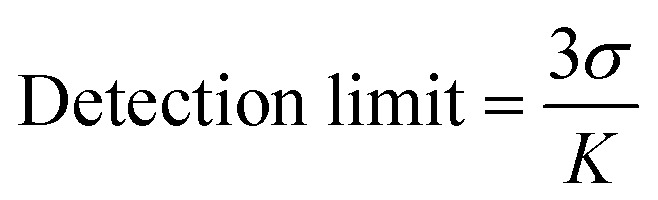


### Bioimaging study

Human embryonic kidney (HEK) 293 cells were maintained in Dulbecco's Modified Eagle's Medium purchased from Sigma Aldrich Inc. and supplemented with 10% fetal bovine serum (FBS) purchased from Atlas biologicals, DMEM – high glucose medium, and antibiotic/antimycotic solution (100×) purchased from Hyclone laboratories.

### Imaging system

Brightfield and fluorescence images were captured at 20× magnification by Leica Microsystems's Leica DMi8 confocal microscope using LAS X software.

### Cell culture

The stock solution of compound 1 was prepared in the mixture of 80% methanol and 20% water (0.1 M HEPES buffer, pH = 7.3) and used for incubation in cells. Cells were adherent-cultured in 8-well culture plates for 72 h. Subsequently, the first group was incubated with probe compound 1 (20.0 mg L^−1^) solution at 37 °C for 40 min, then washed 3 times with phosphate-buffered saline solution (PBS, pH = 7.20). The initial image was acquired using a confocal laser scanning microscope (Leica DMi8) without disturbing the cell positions. The second group was preincubated with probe compound 1 (20.0 mg L^−1^) solution at 37 °C for 40 min, then with DMEM containing zinc acetate (40.0 mg L^−1^) for another 40 min, and then washed with PBS for 3 times. These cells were underwent imaging measurement by a confocal microscope (Leica DMi8). The exciting light was 410 nm. The emission range of the blue channel was 420–600 nm.

## Results and discussion

### Synthesis and characterization

The synthesis of 1 was carried out in two different solvent systems, aqueous and organic solvent (CH_3_OH), as shown in Scheme 1, and leads to the same product. In aqueous solvent, the pyridoxal hydrochloride was first neutralized with NaHCO_3_ to pH = 7 using pH indicator strips; whereas, in an organic solvent, it is neutralized with triethylamine. In either case, the colorless solution turned yellow in color, and the yield of the product was above 84%. The final product, compound 1, was recrystallized by slow evaporation in a mixture of methanol and ether and characterized using ^1^H NMR, ^13^C NMR, COSY NMR, CHN analyses, ESI-MS, and X-ray crystallography.

^1^H NMR of the free ligand (1, Fig. S1†) was carried out using DMSO-*d*_6_ as a solvent. The ^1^H NMR spectra of 1 showed a singlet at 8.89 ppm due to the presence of the CH=N– linkage, which confirms the formation of the Schiff-base. In the free ligand, the phenolic proton has been transferred to the imine nitrogen to form the zwitterion, which has also been confirmed by single-crystal X-ray crystallography (below). A singlet zwitterion proton on the imine nitrogen appears at 15.44 ppm (donated from the nearby phenol group) and the carbimine proton appears at 8.89 ppm and are coupled to each other as indicated by COSY NMR in Fig. S3.† The aromatic proton from the pyridine ring appears at 7.72 ppm. The triplet peaks at 5.32 ppm and 4.89 ppm displayed the presence of the aliphatic –OH groups and the doublet peaks at 4.60 ppm and 3.63 ppm displayed the aliphatic methylene –CH_2_ groups from the pyridoxal and TRIS moieties, respectively, with identical coupling for the –CH_2_–OH pairs. The singlet peak at 2.34 ppm displays the presence of the methyl group from the pyridoxal ring.

The ^13^C NMR spectra (Fig. S4†) of 1 displayed a sharp characteristic peak due to the azomethine carbon, which resonates at 162.48 ppm. The signal observed at 19.34 ppm belongs to the methyl group. The signals observed at 117.89, 133.07, 134.99, 150.76, and 158.75 ppm are assigned due to pyridoxal ring carbon moieties. The signal observed at 68.02 ppm is due to the quaternary carbon and the signals observed at 58.96 and 61.54 ppm are due to the –CH_2_ groups. The ^13^C-DEPT NMR reveals positive signals at 135.0 ppm (aromatic –CH) and 19.3 ppm (aliphatic –CH_3_) and negative signals for 58.9 and 61.6 ppm (aliphatic –CH_2_ groups for pyridoxal and TRIS).

### Optical properties

**Fig. 1:**
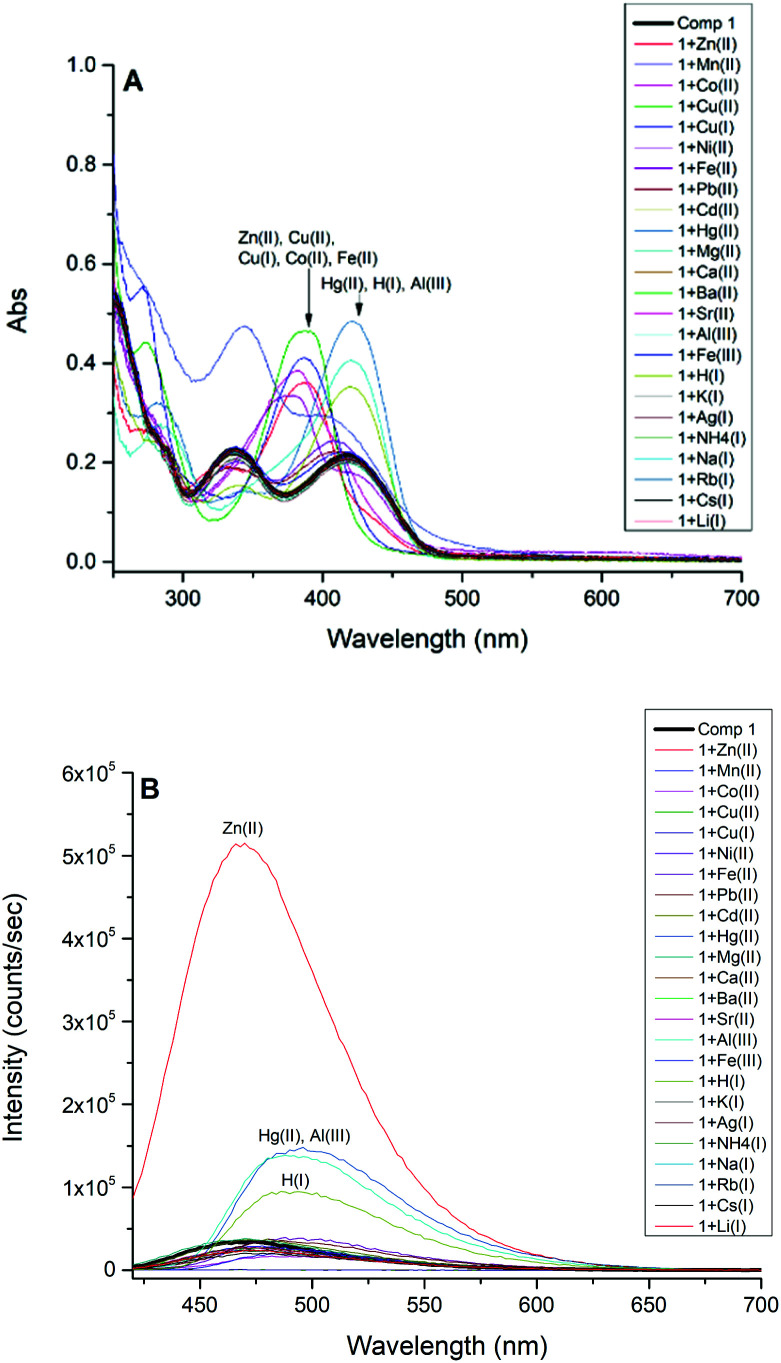
(A) UV-Vis spectra of compound 1 (5.0 × 10^−5^ M). (B) Emission spectra of compound 1 (5.0 × 10^−5^ M, *λ*_ext_ = 413 nm) in CH_3_OH before (black) and after the addition of 2.0 equivalents of cations.

The absorbance spectra of 1 in methanol displayed two distinctive absorbance bands centered at 277 nm and 329 nm, which are assigned to π–π* transitions, and a third absorbance peak at 420 nm attributed to n–π* transition due to the azomethine group. However, the addition of 2.0 equivalents of Zn(ii), Co(ii), Cu(ii), Cu(i), Fe(ii), Hg(ii), Al(iii), Fe(iii), and H(i) induced dramatic bathochromic shifts to longer wavelengths, indicating the formation of ligand–metal adducts with 1 (Fig. 1A). The fluorescence activity of 1 was also examined by adding 2.0 equivalents of metal salts and collecting the resulting emission spectra (Fig. 1B), indicating high selectivity for Zn(ii) ion which exhibits an intense fluorescent band at 470 nm. The addition of Cu(ii) quenched the fluorescence intensity of 1 completely. The addition of H(i), Hg(ii), and Al(iii) have moderate fluorescence enhancements but are not biocompeting cations under physiological conditions.

**Fig. 2:**
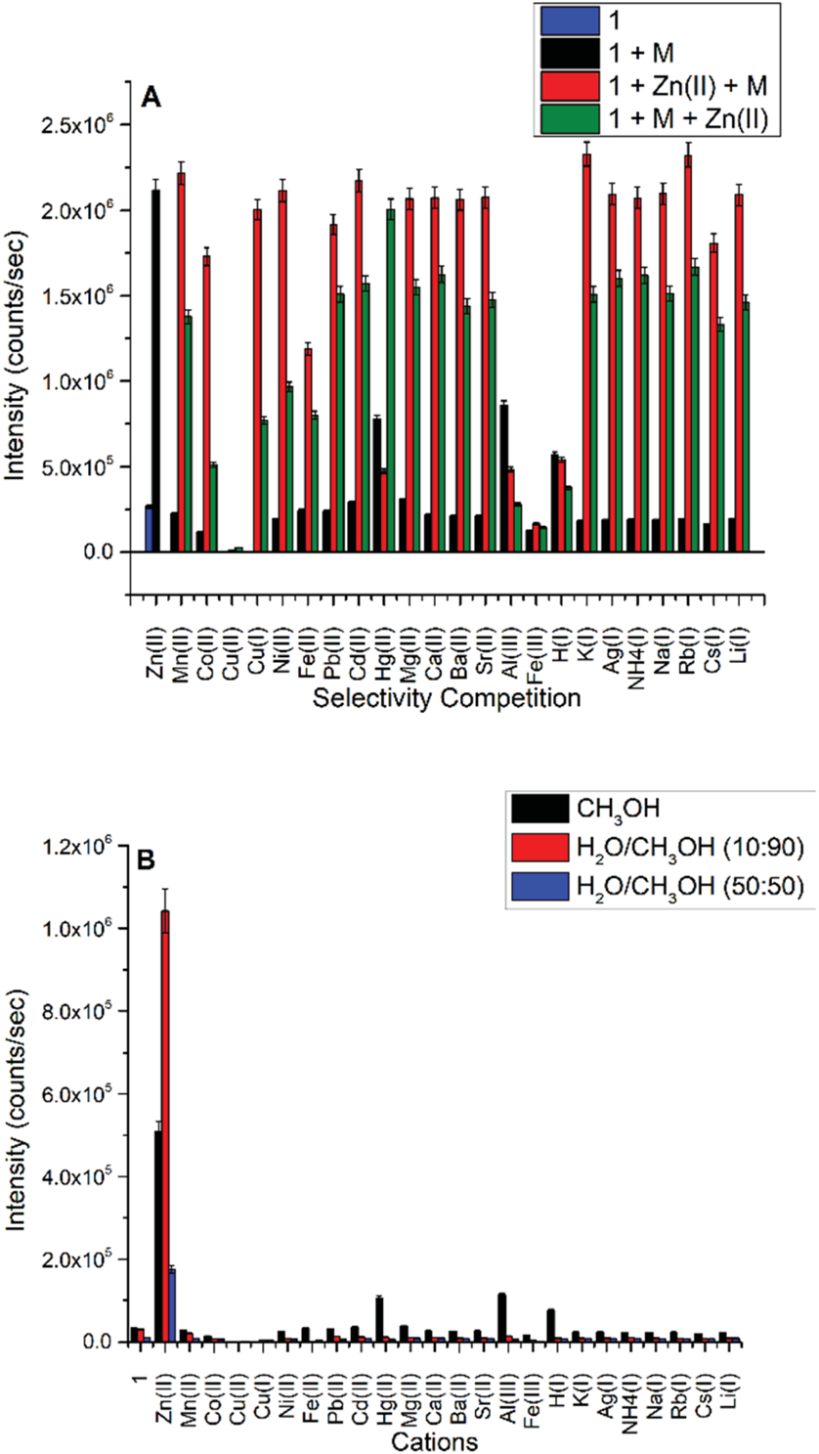
(A) Selectivity – competition study of 1 with addition of 2.0 equivalents of cations at 470 nm. (B) Selectivity based on the fluorescence intensity profile of 1 with addition of 2.0 equivalents of cations in the different solvent systems at 470 nm. *λ*_ext_ = 413 nm. H_2_O contains 0.1 M HEPES buffer, pH = 7.3.

A competition study for Zn(ii) (Fig. 2A) with the same cations shows that Zn(ii) selectivity has increased ∼6 fold over the ligand, and the luminescence, for the most part, is maintained for Zn(ii) even in the presence of most of the cations, except for Cu(ii), Al(iii), Fe(iii), Hg(ii), and H(i). In the case of Cu(i) and Cu(ii), both quenched the weakly fluorescent 1; however, only Cu(ii) can quench the fluorescence of 1 in the presence of Zn(ii).

Subsequently, we studied the selectivity of compound 1 towards Zn(ii) in different solvent systems (Fig. 2B), including 100% methanol, 90% methanol/10% water (0.1 M HEPES buffer, pH = 7.3), and 50% methanol/50% water (0.1 M HEPES buffer, pH = 7.3). The Schiff base is hydrolyzed by water, hence we tested the fluorescence intensity to detect Zn(ii) in different fractions of an aqueous (0.1 M HEPES buffer, pH = 7.3) mixture of CH_3_OH and DMSO presented in Fig. S16 and S17† respectively which suggest the loss of emission intensity at higher content of water.

The selectivity of Zn(ii) in all studied solvent systems is maintained with the highest sensitivity/intensity exhibited using HEPES buffer, potentially due to pH differences between these buffered and unbuffered solvents. Enhanced fluorescence due to H(i), Hg(ii), and Al(iii) cations were not observed in the other solvent systems, only in methanol. Compound 1 is highly selective and sensitive towards Zn(ii) ion, and acts as a fluorometric and colorimetric sensor with large fluorescence enhancement in the presence of Zn(ii), and the color of 1 (yellow in CH_3_OH) turns green in the presence of Cu(ii), red in the presence of Co(ii), and burgundy in the presence of Mn(ii).

### pH-dependence and reversibility

The enhanced fluorescence response of 1 with Zn(ii) in 10% H_2_O (0.1 M, HEPES buffer, pH = 7.3) revealed that compound 1 may be pH-dependent. Fig. S10† shows that fluorescence intensity maximizes at pH = 7 and quenches under acidic and basic conditions. We have also studied the UV-Vis and fluorescence responses of 1 with added anions, and relative nondescript spectral changes occur with different strength anionic bases, as shown in Fig. S11 and S12† as well. In addition, reversible binding of guests is a defining principle of chemosensors. Hence, the reversibility of Zn(ii) binding with 1 in methanol was investigated using the strong chelating ligand EDTA. The addition of an aqueous solution of EDTA quenched the enhanced fluorescence response of compound 1 with Zn(ii) which reappeared with the following addition of Zn(ii), as shown in Fig. S8,† supporting the binding of Zn(ii) with 1 as reversible. The ESI-MS spectrum of recovered 1 after the addition of an aqueous solution of EDTA shows the molecular ion peaks (*m*/*z*) of 271.1 [M + H]^+^ as shown in Fig. S9.†

### Binding constant determination

**Fig. 3:**
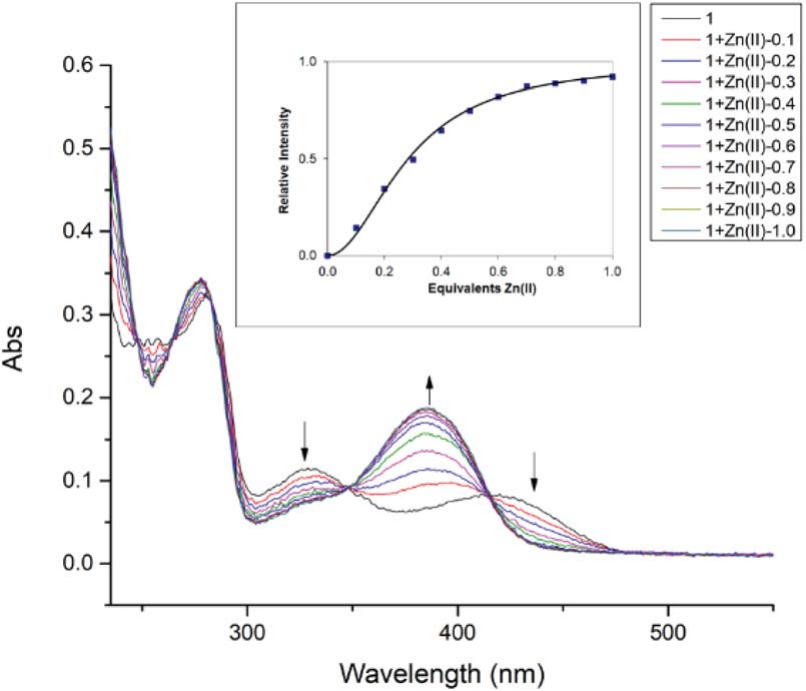
Changes in the UV-Vis spectra of compound 1 in CH_3_OH upon addition of Zn(ii) in CH_3_CN. Inset: relative absorbance intensity at 385 nm with added equivalents of Zn(ii) and best fit for 2 : 1 1 : Zn(ii) association. The association constant for a 2 : 1 best fit equals 1.2 × 10^9^ M^−2^ (±25%).

To further investigate the interaction between compound 1 with Zn(ii), the UV-Vis titration of 1 with an increasing amount of added Zn(ii) was carried out in methanol, until the absorbance and fluorescence intensities saturate as shown in Fig. 3 and S15† respectively. The absorption peaks at 329 nm and 420 nm gradually decrease, giving a new strong absorption peak at 385 nm, possibly related to phenolate (O–) and Zn(ii) coordination. Similar spectral behavior was observed between 240–390 cm^−1^ for the perturbation of ligand moieties (–(OH)C=C–C=N–) due to the coordination of Zn(ii) during the reaction period.^31^ These spectral changes are also accompanied by clean isosbestic points at 265 nm, 282 nm, 348 nm, and 413 nm, which indicates the formation of a single zinc complex. The change in absorption at 385 nm was used to calculate the binding constant as shown in Fig. 3 (inset). The data points (squares) follow a sigmoidal curve for 2 : 1 complexation of compound 1 with Zn(ii), with saturation occurring at approximately 0.5 equivalents of added Zn(ii). The 2 : 1 association constant represents the equilibrium 2L + Zn(ii) ↔ L_2_Zn(ii) and equals 1.2 × 10^9^ M^−2^ (±25%) resulting in the best fit.^80^ The inflection point in Job's plot (Fig. S19†) *via* fluorescence titration at the mole fraction of ∼0.33 further confirms the formation of the L_2_Zn(ii) complex.

### Quantum yield and detection limit

The quantum yield of compound 1 and its Zn(ii) complex at *λ*_ex_ = 377 nm in ethanol (referenced to anthracene as standard, *ϕ* = 0.27 in ethanol), produced a quantum yield that was ∼12 times larger in the presence of Zn(ii) ion (*ϕ* = 0.48) compared to the compound 1 (*ϕ* = 0.04). These values are comparable to the quantum yields of the pyridoxal derivatives previously reported.^31^ The titration between 1 and Zn(ii) results in a linear equation *y* = 260 29*x* + 5649.5 (*R*^2^ = 0.992) in the range of 5–25 × 10^−6^ M concentration of Zn(ii) at 472 nm as shown in Fig. S13† and the detection limit was found to be 2.8 × 10^−7^ M (28 μM) which is observable below the World Health Organizations guideline (76.5 μM) for Zn(ii) in water.^71^

### Crystallography

Single crystals of 1, the Zn(ii) complex, and a related Cu(ii) complex were selected for X-ray crystallographic analysis, and data were collected on a Bruker D8 Venture at 100 K. Crystal system, unit cell, space group, and other parameters are presented in Table 1.

**Table 1:** Crystallographic data table showing important parameters such as unit cell, crystal system, space group, *etc.*

	Compound 1	[1^−^]_2_Zn^2+^·5H_2_O	[1]Cu^2+^·2ClO_4_^−^
Empirical formula	C_12_H_18_N_2_O_5_	C_24_H_44_N_4_O_15_Zn	C_12_H_18_N_2_O_13_Cl_2_Cu_1_
Formula weight	270.29	694.02	532.72
Temperature (K)	100	100	100
Crystal system	Triclinic	Orthorhombic	Triclinic
Space group	*P*1̄	*Pna*2_1_	*P*1̄
Unit cell (Å, °)	*a* = 7.5493(3)	*a* = 20.3659(17)	*a* = 5.8767(23)
	*b* = 9.2659(3)	*b* = 8.7855(9)	*b* = 9.1251(39)
	*c* = 9.8603(3)	*c* = 16.9217(19)	*c* = 18.2139(84)
	*α* = 91.084(2)	*α* = 90	*α* = 86.630(17)
	*β* = 104.586(1)	*β* = 90	*β* = 80.725(15)
	*γ* = 109.587(1)	*γ* = 90	*γ* = 74.305(14)
Volume (Å^3^)	624.83(4)	3027.70(5)	927.90(4)
*Z*	2	4	2
Density diffraction	1.44	1.522	1.91
Absorption coefficient	0.946	0.889	1.540
*F*(000)	288.0	1464.0	542.0
*θ* range	4.7–74.3	2.0–25.4	2.3–26.4
Index ranges	±9 ± 11 ± 12	±24 ± 10 ± 20	±7 ± 11 ± 22
Reflection collected	19 191	23 510	24 629
Independent reflections	2534	5476	3771
Observed reflections	2269	3725	2240
Goodness-of-fit (GOOF)	1.112	1.045	1.031
Final *R* indices [*I* > 2*σ*(*I*)]	0.049	0.058	0.079
*R* indices (all data)	0.053	0.101	0.139
CCDC deposit number	2 095 325	2 095 330	2 095 332

**Fig. 4:**
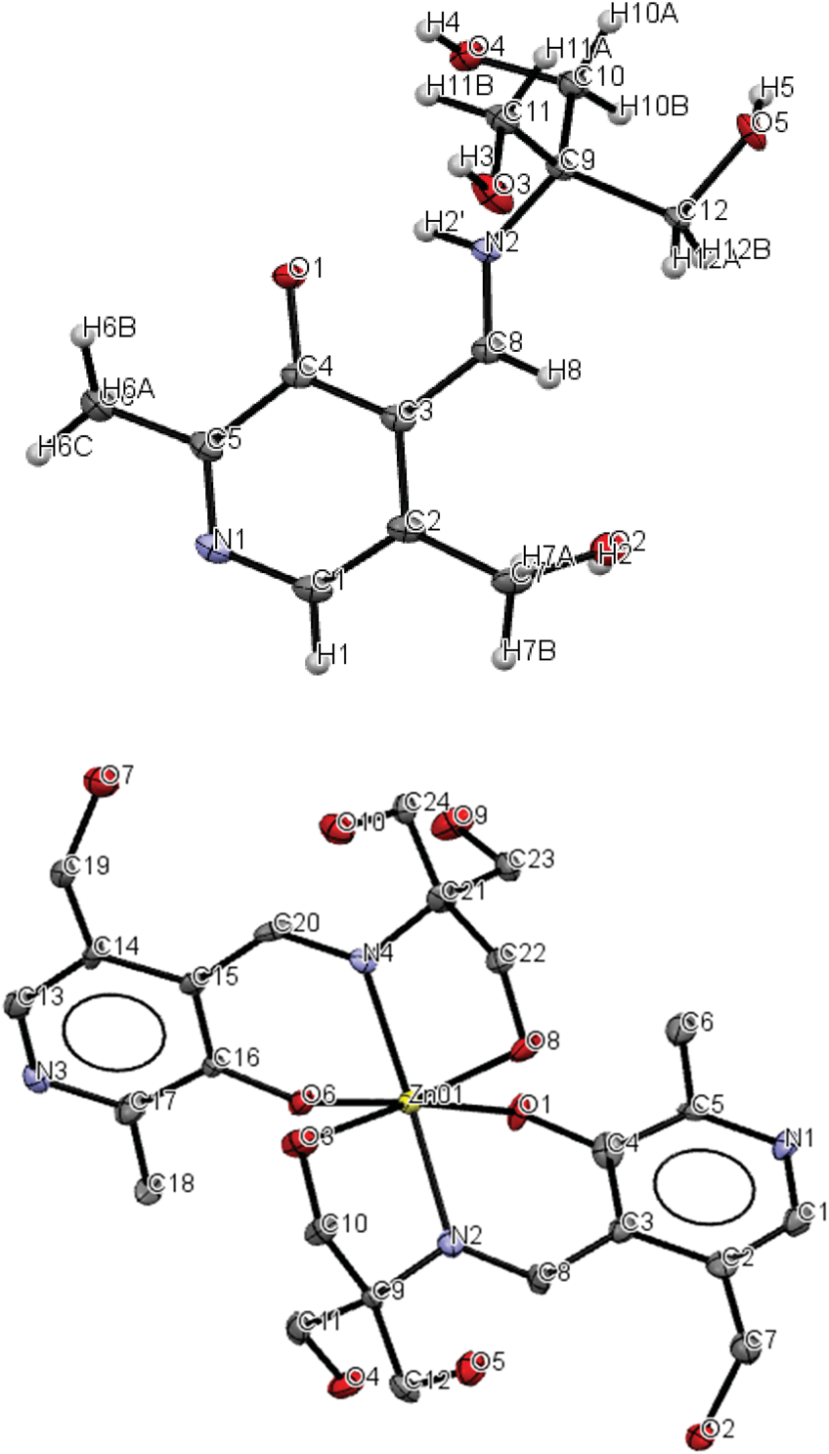
Thermal ellipsoid diagram (50%) of compound 1 and its Zn(ii) complex. (Grey: carbon, red: oxygen, purple: nitrogen, yellow: zinc.) Hydrogen atoms, disordered atoms, and water molecules are not shown for clarity.

Compound 1 adopts a zwitterion structure (see Fig. 4) with the proton transferred from the pyridoxal phenol to the imine nitrogen (N_2_). The zwitterion structure is generally observed in pyridoxal-containing derivatives; however, in this case, the imine nitrogen is protonated instead of the nitrogen atom of the pyridoxal ring.^72^ The bond angles and distances presented in Table 2 are in good agreement with those compounds where the zwitterion is adopted.^73^ The C8–N2–C9 angle (127.29°) is significantly greater than the normal imine bond angle and is characteristic of the protonated nitrogen. This is also in agreement with the C4–O1 distance (1.302 Å), which is intermediate between C=O distance (1.23 Å) and C–O distance (1.43 Å), and O1–N2 distance (2.587 Å), a suitable distance for intramolecular hydrogen bonding. The C and O atoms of one alkoxy group at C12 are disordered with equal occupancies.

**Table 2:** Important bond lengths and angles of different connectivity in the crystal structure

	Compound 1	[1^−^]_2_Zn^2+^·5H_2_O	[1]Cu^2+^·2ClO_4_^−^
C4–O1	1.302	1.332	1.280
C8–N2	1.287	1.282	1.271
N2–C9	1.471	1.472	1.494
C3–C2	1.419	1.432	1.436
C3–C4	1.416	1.412	1.416
C1–C2	1.370	1.352	1.386
C1–N1	1.357	1.361	1.359
N1–C5	1.327	1.332	1.324
C5–C4	1.443	1.412	1.433
C8–N2–C9	127.29	120.55	127.1
C3–C4–O1	123.07	123.67	126.16
C5–C4–O1	120.19	118.20	116.63
C1–N1–C5	119.78	118.10	125.46

We report the crystal structures of the Zn(ii) and Cu(ii) complexes of compound 1 to gain comprehensive knowledge of the structural variations of these two metal complexes. As shown in the crystal structure in Fig. 4, the reaction between 1 and hydrated zinc perchlorate in either 1 : 1 or 2 : 1 ratios generated crystals of a complex containing two molecules of 1 and one zinc(ii) ion, confirming the previous 2 : 1 stoichiometry. Five water molecules were also found in the structure, hydrogen-bonded to the hydroxyl and nitrogen-containing moieties. The center zinc ion is coordinated through the imine nitrogen and the phenolate and alkoxide oxygen atoms from each of the two ligands, achieving a distorted six-coordinate octahedral geometry. The two deprotonated phenoxy oxygen atoms and two protonated alkoxy oxygen atoms occupy the equatorial position, whereas the two imino nitrogen atoms adopt an axial, trans arrangement. The angle between the mean planes through the pyridine rings of the two ligands in Zn-complex is 78.36°. Other fluorescent probes have been reported which bind with Zn(ii),^74^ Cu(ii),^75,76^ and Co(ii),^77^ and a similar structure ((6-methoxy-2-[tris-(hydroxymethyl)-methyl-imino-methyl]-phenolate), incorporating benzene instead of pyridine) to 1 has been reported by Zhang *et al.*, making a complex with Mn(ii).^78^

**Fig. 5:**
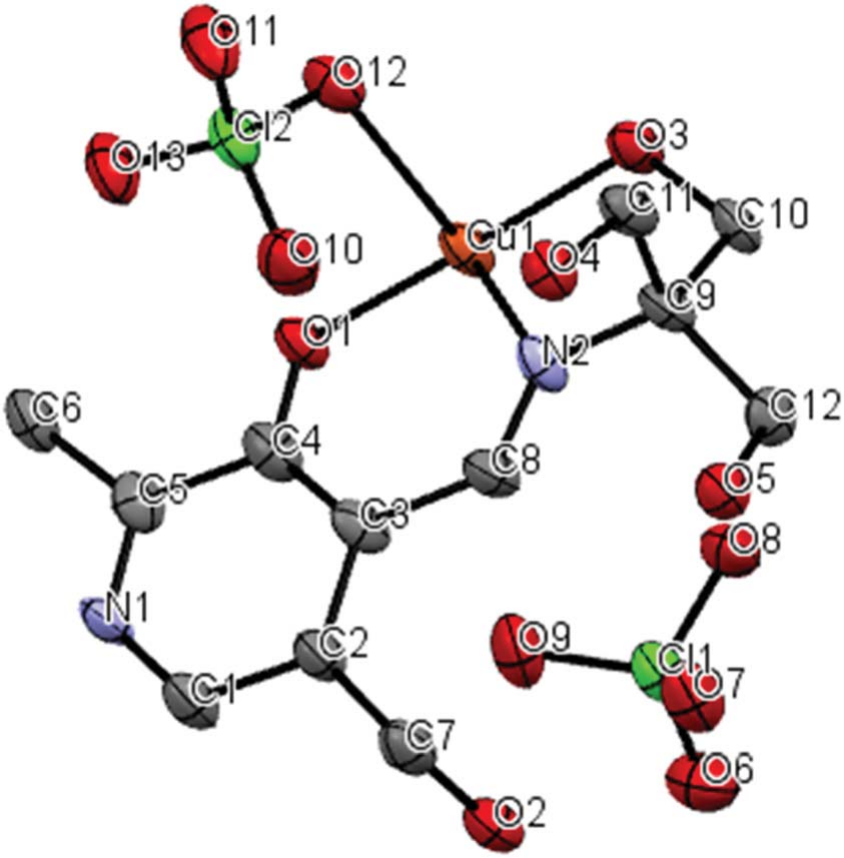
Thermal ellipsoid diagram (50%); asymmetric unit of copper complex, hydrogen atoms were not shown for clarity.

**Fig. 6:**
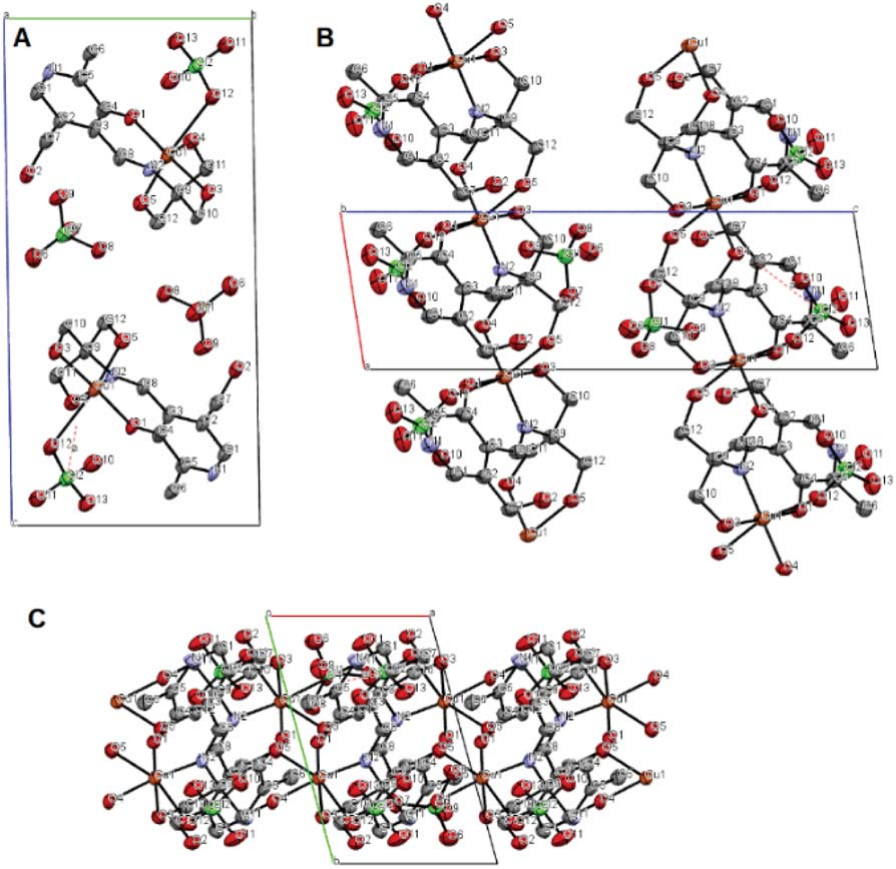
Ortep diagram of the polymeric form of 3 units of [1]Cu^2+^·2ClO_4_^−^, hydrogen atoms were not shown for clarity, (A) along the *a*-axis, (B) along the *b*-axis, and (C) along the *c*-axis. (Grey: carbon, red: oxygen, purple: nitrogen, orange: cupper, and green: chlorine.)

A crystal structure of the corresponding copper(ii) complex reveals a different 1-dimensional polymeric structure of the building-block shown in Fig. 5. In this case, 1 forms a 1 : 1 complex with Cu(ii) ion; one molecule of 1 chelate the Cu(ii) ion with the imine nitrogen, one phenolic oxygen, and one alkoxy oxygen, combined with two alkoxy oxygen atoms from a neighboring complex, plus one perchlorate ion to form a coordination polymer with distorted six-coordinate octahedral geometry, *i.e.* each ligand is linked to two copper centers through different parts of the molecule, propagating along the *a*-axis (Fig. 6). The pyridine rings of two different ligands are parallel and the distance between them is 3.33 Å. We speculate that Cu(ii) ion is more oxophilic and binds to the extra alkoxy groups of the TRIS rather than to another imine functional group.

### *In vitro* cell studies of compound 1

**Fig. 7:**
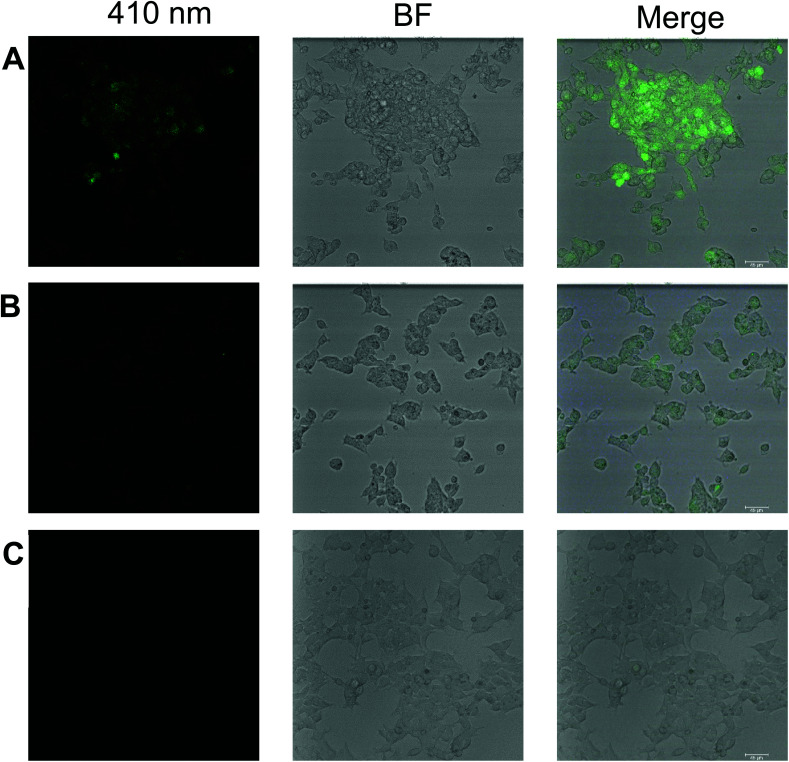
The fluorescence (410 nm) and brightfield (BF) images of human embryonic kidney cells referred as HEK293 or HEK cells; (A) preincubated with 20 ppm compound 1 at 37 °C for 40 min and then incubated with 40 ppm of zinc acetate at 37 °C for another 40 min, (B) incubated with 20 ppm of compound 1 at 37 °C for 40 min, and (C) before incubation; scale: 45 μm.

We believe that the outstanding turn-on response, sensitivity, selectivity, low detection limit, and reversibility of compound 1 toward zinc ion can find a practical application in biological cell imaging. In this regard, we studied the *in vitro* fluorescence response of 1 to detect zinc ion in human embryonic kidney cells referred as HEK293 or HEK cells. In this study, HEK293 cells are incubated with 20 ppm 1 alone and 1 plus 40 ppm Zn(ii), and compared to cells alone using confocal microscopy with corresponding brightfield and fluorescence images with an objective of 20×. Here we find that even without preincubating with Zn(ii), compound 1 exhibits weak fluorescence with living cells as shown in Fig. 7B, and a stronger fluorescence signal is observed when 40 ppm zinc ion is incubated along with 20 ppm 1 as shown in Fig. 7A as monitored using the 410 nm blue excitation channel. Cells without 1 and Zn(ii) do not show fluorescence, Fig. 7C. The mean fluorescence intensity (MFI) was calculated according to the method described previously^79^ by using Fiji software as shown in Fig. S18† which suggests the MFI of compound 1 with Zn(ii) is increased ∼5 fold over compound 1 in HEK293 cells. Previously, pyridoxal-based chemosensors were used for bio-imaging A549, human lung cancer cells, by fluorescence microscopy, but only exhibited fluorescence when incubated together with added zinc ion.^31^ This suggests that 1 has the potential to be used in incubated cells spiked with Zn(ii).

## Conclusion

The synthesis of compound 1 and the resulting Zn(ii) and Cu(ii) metal complexes were carried out and fully characterized by standard analytical techniques. Compound 1 was found to be highly selective and sensitive towards Zn(ii) in aqueous solution *via* fluorescence turn-on response. Zn(ii) ion titrations and the crystal structure of the Zn(ii) complex showed the formation of a 2 : 1 complex with Zn(ii) with a distorted octahedral geometry and a high binding affinity. The crystal structure of the copper complex, the only major competing cation, reveals a different 1 : 1 binding motif and the formation of a coordination polymer instead. The bio-imaging of compound 1 was conducted using HEK 293 cells *via* blue-channel fluorescence at 410 nm, and even before incubating with added Zn(ii) ion, weak fluorescence of these cells was observed. We believe that the outstanding turn-on response and sensitivity toward zinc ion will find future applications in chemical and biological science.

## Conflicts of interest

The authors declare that they have no known competing financial interests or personal relationships that could have appeared to influence the work reported in this paper.

## Supplementary Material

RA-011-D1RA05763D-s001

RA-011-D1RA05763D-s002
