# Supplementary material for: A highly selective pyridoxal-based chemosensor for the detection of Zn(ii) and application in live-cell imaging; X-ray crystallography of pyridoxal-TRIS Schiff-base Zn(ii) and Cu(ii) complexes
Source: RSC Adv. 2021 Oct 21;11(54):34181–92. doi: 10.1039/d1ra05763d (PMC9042327; doi:10.1039/d1ra05763d)
Supplement: RA-011-D1RA05763D-s001 [file RA-011-D1RA05763D-s001.pdf]

Electronic Supplementary Material (ESI) for RSC Advances.
This journal is © The Royal Society of Chemistry 2021

Supporting Information

# A highly selective pyridoxal-based chemosensor for the detection of Zn(II) and application in live cell imaging; X-ray crystallography of pyridoxal-TRIS Schiff-base Zn(II) and Cu(II) complexes.

Anwar Hussain,[a] Kadarkaraisamy Mariappan,[a] Dawson C. Cork,[a] Luke Lewandowski,[a] Prem K. Shrestha,[a] Samiksha Giri,[b] Xuejun Wang,[b] and Andrew G. Sykes*[a]

[a] *Contribution from the Department of Chemistry, University of South Dakota, Vermillion, SD 57069, United States of America.*
[b] *Basic Biomedical Sciences, University of South Dakota School of Medicine, Vermillion, SD 57069, United States of America.*

*Corresponding author: Email: Andrew.Sykes@usd.edu*

Figure S1: $^{1}H$ NMR of compound 1 in DMSO-d6........2
Figure S2: $^{1}H$ NMR of compound 1 in DMSO-d6 with added $D_2O$. ........2
Figure S3: $^{1}H$-$^{1}H$ Correlation Spectroscopy (COSY) NMR of 1 shows the correlation between hydrogens coupled to each other in the $^{1}H$ NMR spectrum in DMSO-d6. ........3
Figure S4: $^{13}C$-CPD NMR of compound 1 in DMSO-d6. ........3
Figure S5: $^{13}C$-DEPT135 NMR of 1 in DMSO-d6. ........4
Figure S6: IR spectrum of compound 1. ........4
Figure S7: ESI-MS Spectra of **1** showing a molecular ion peak at 271.2 $[M+H]^+$ referring to the molar mass of compound **1** (270.28 g/mol). ........5
Figure S8: Fluorescence emission spectra of compound 1 in the presence of Zn(II) followed by addition of $Na_2EDTA$ and followed by the addition of Zn(II) (λex=413 nm). ........5
Figure S9: ESI-MS Spectra of recovered **1** after addition of EDTA showing a molecular ion peak at 271.1 $[M+H]^+$ referring to the molar mass of compound **1** (270.28 g/mol). ........6
Figure S10: Effect of the pH on the fluorescence intensity of compound 1 in the presence of 2 equivalent of Zn(II) ions (λex=413 nm). ........6
Figure S11: UV-Vis spectra of compound **1** (5.0 $\times 10^{-5}$ M) in $CH_3OH$ before (black) and after the addition of 2.0 equivalents of anions as tetrabutylammonium salts. ........7
Figure S12: Emission spectra of compound **1** (5.0 $\times 10^{-5}$ M, $\lambda_{ext}$ = 413 nm) in $CH_3OH$ before (black) and after the addition of 2.0 equivalents of anions as tetrabutylammonium salts. ........7
Figure S13: Normalized response of fluorescence signal of 1 ($5.0\times10^{-5}$ M, $CH_3OH$) in the presence of the increasing amount of Zn(II) (5 – 25 $\times10^{-6}$ M, ACN) ($\lambda_{ex}$ = 413nm; $\lambda_{em}$ = 470nm) ........8
Figure S 14: Electrospray mass spectrum (ESI-MS positive) of Cu(II) complex of compound **1**. ........8
Figure S15: Relative fluorescence intensity at 470 nm on the added equivalent of Zn(II); best fit for 2:1 compound 1:Zn(II) association. Association constant for 2:1 best fit equal 6.0 x $10^{9}$ $M^{-2}$ (±10%). ........9
Figure S16: Relative fluorescence intensity of compound 1 at 470 nm on addition of 2.0 equivalents Zn(II) as perchlorate salts in different fraction of $CH_3OH$ and $H_2O$ (0.1 M HEPES buffer, pH = 7.3) showing the loss of emission at higher content of water. 20 scans with interval of 1 min, $10^{th}$ scan: approx. 15 mins, and $20^{th}$ scan: approx. 30 mins. ........9
Figure S17: Relative fluorescence intensity of compound 1 at 470 nm on addition of 2.0 equivalents Zn(II) as perchlorate salts in different fraction of DMSO and $H_2O$ (0.1 M HEPES buffer, pH = 7.3) showing the loss of emission at higher content of water. 20 scans with interval of 1 min, $10^{th}$ scan: approx. 15 mins, and $20^{th}$ scan: approx. 30 mins. ........10
Figure S18: Mean fluorescence intensity(MFI) of compound 1 in HEK293 cells calculated using Fiji software. ........10
Figure S19: Job's plot of **1** with Zn(II). Fluorescence intensity monitored at 470 nm vs. a function of the molar ratio of [**Zn(II)**]/([**1**] + [**Zn(II)**]), $\lambda_{ex}$ = 413nm. ........11

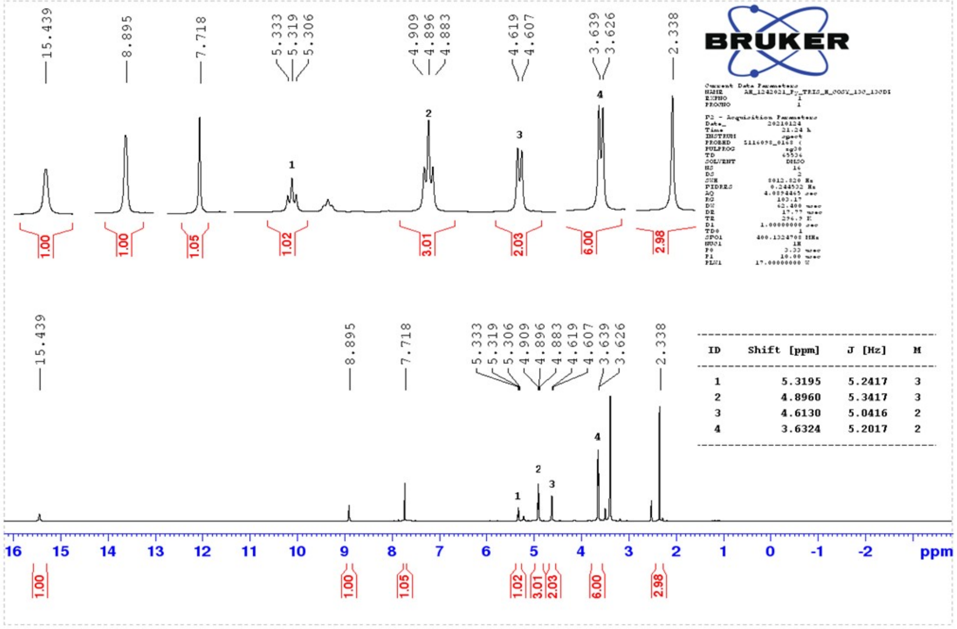


*Figure S1: $^{1}$H NMR of compound 1 in DMSO-d6.*

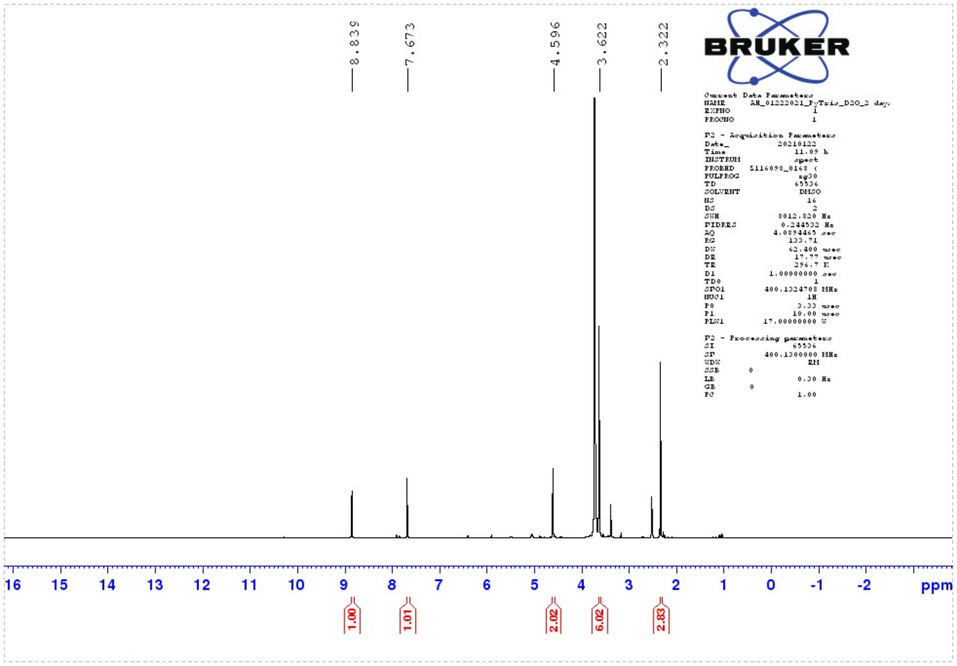


*Figure S2: $^{1}$H NMR of compound 1 in DMSO-d6 with added $D_2O$.*

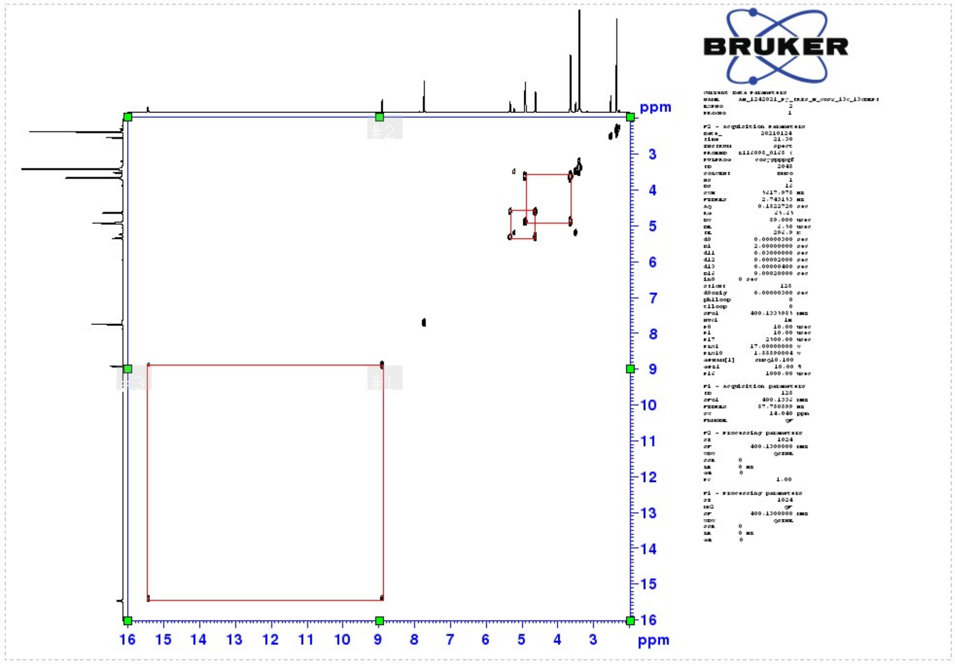


*Figure S3: ¹H-¹H Correlation Spectroscopy (COSY) NMR of 1 shows the correlation between hydrogens coupled to each other in the ¹H NMR spectrum in DMSO-d6.*

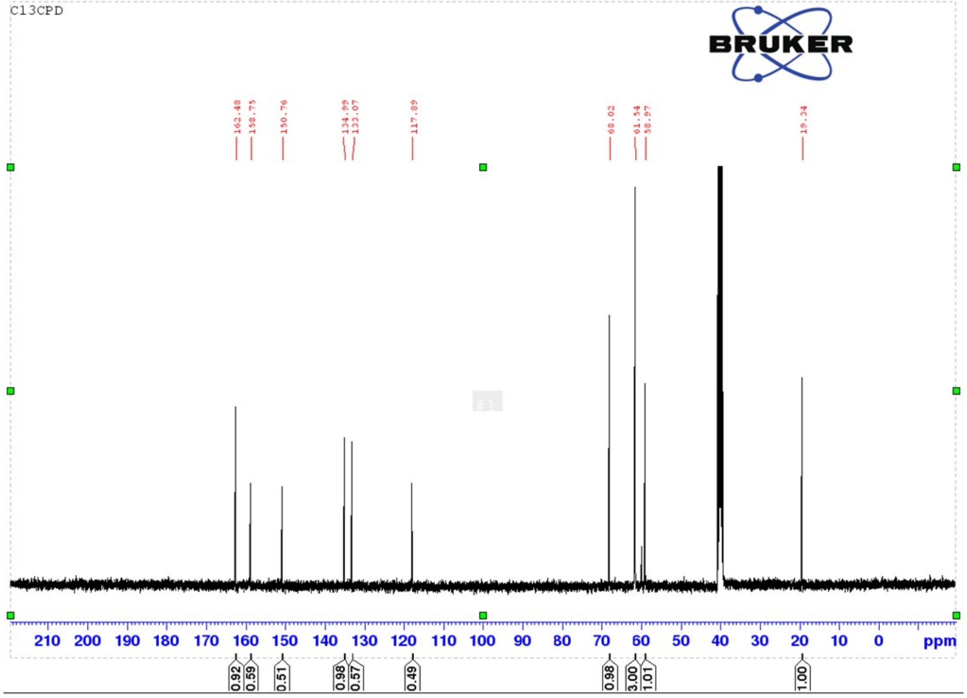


*Figure S4: ¹³C-CPD NMR of compound 1 in DMSO-d6.*

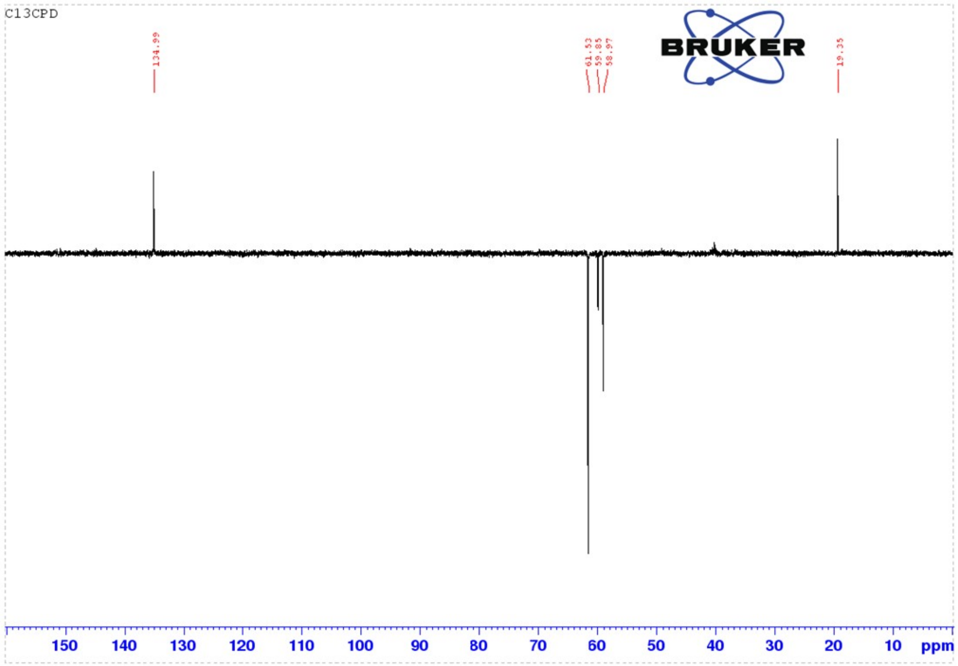


*Figure S5: $^{13}$C-DEPT135 NMR of 1 in DMSO-d6.*

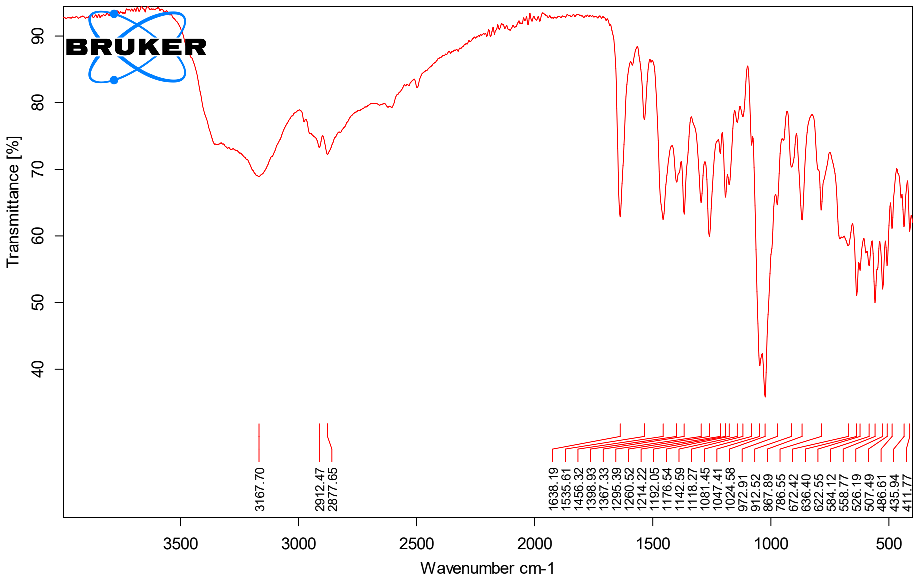


*Figure S6: IR spectrum of compound 1.*

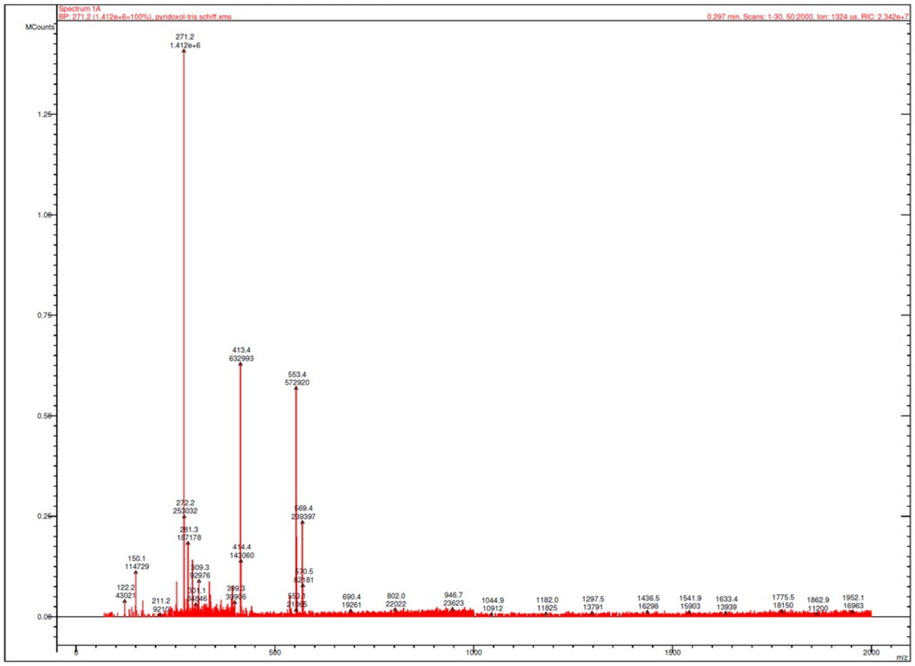

*Figure S7: ESI-MS Spectra of **1** showing a molecular ion peak at 271.2 $[M+H]^+$ referring to the molar mass of compound **1** (270.28 g/mol).*

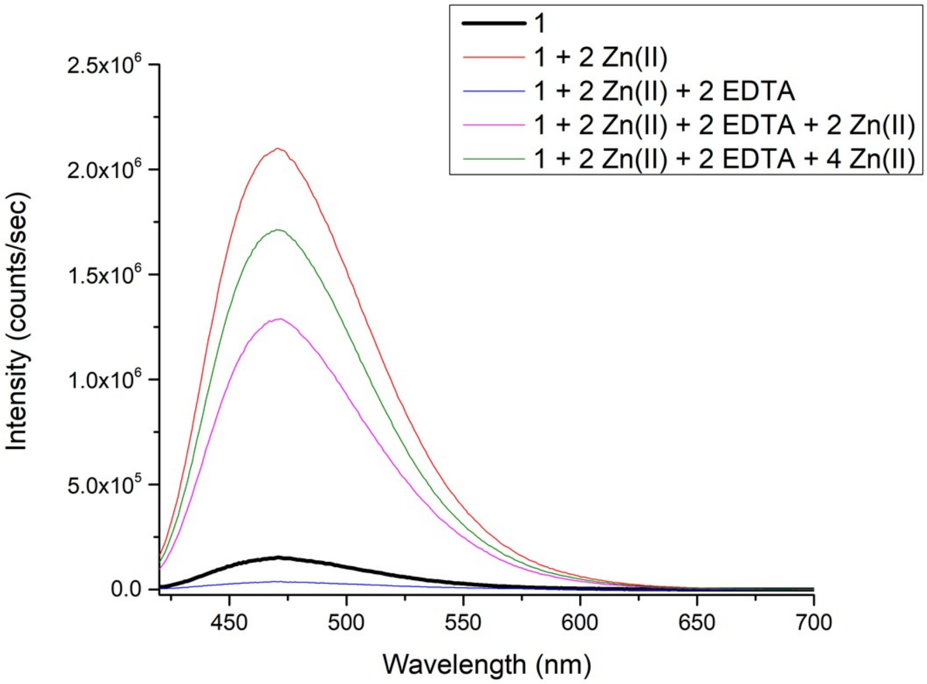


*Figure S8: Fluorescence emission spectra of compound 1 in the presence of Zn(II) followed by addition of $Na_2EDTA$ and followed by the addition of Zn(II) (λex=413 nm).*

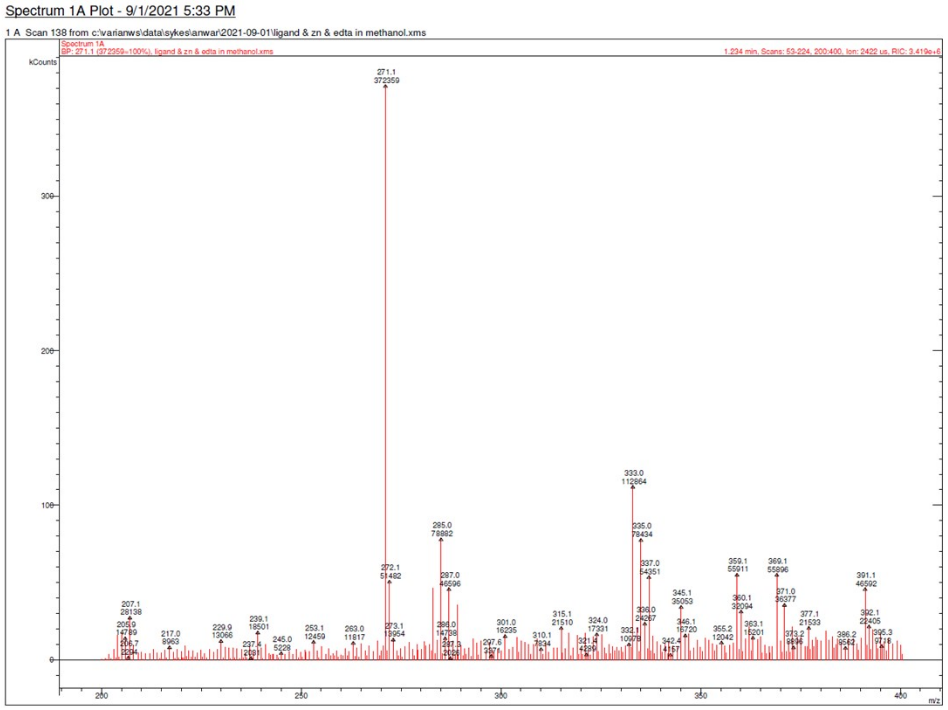


*Figure S9: ESI-MS Spectra of recovered **1** after addition of EDTA showing a molecular ion peak at 271.1 $[M+H]^+$ referring to the molar mass of compound **1** (270.28 g/mol).*

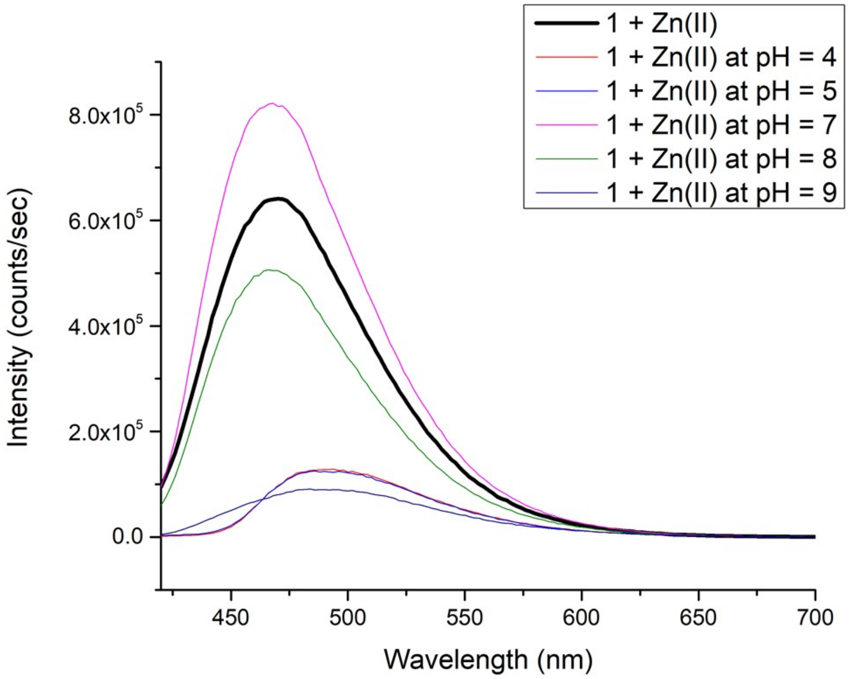


*Figure S10: Effect of the pH on the fluorescence intensity of compound 1 in the presence of 2 equivalent of Zn(II) ions (λex=413 nm).*

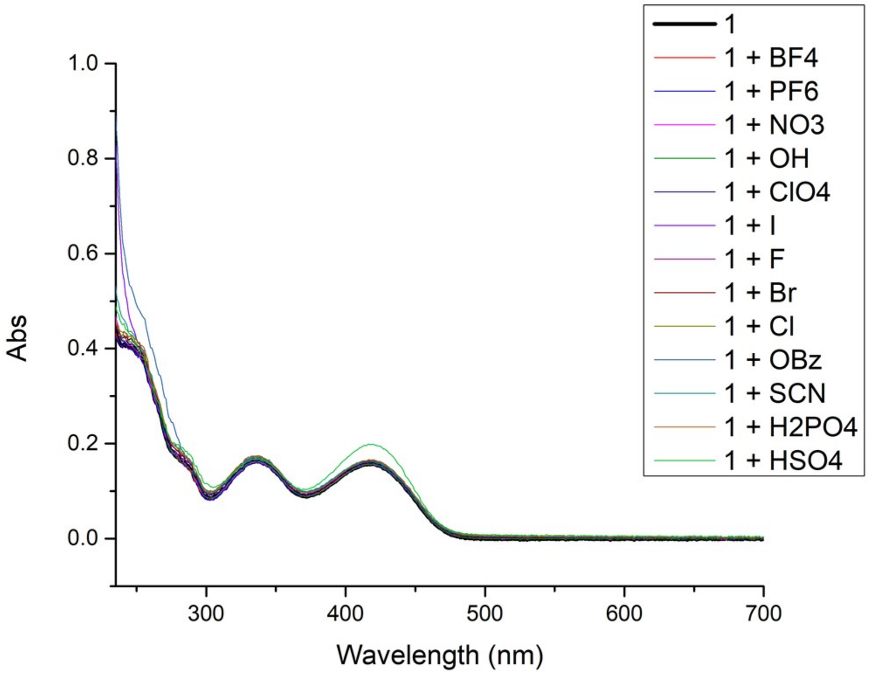


*Figure S11: UV-Vis spectra of compound **1** ($5.0 \times 10^{-5}$ M) in $CH_3OH$ before (black) and after the addition of 2.0 equivalents of anions as tetrabutylammonium salts.*

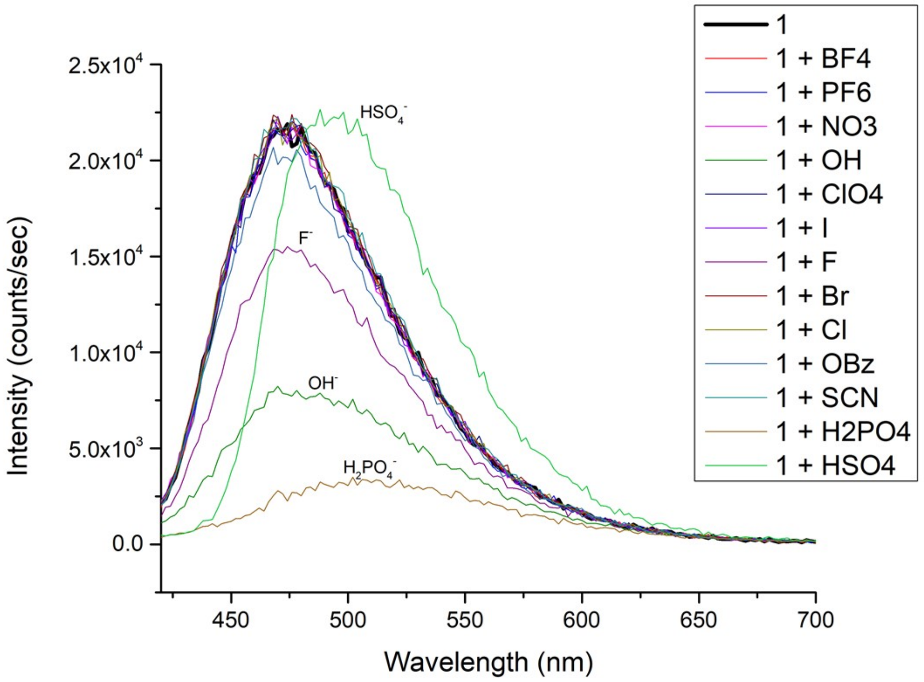


*Figure S12: Emission spectra of compound **1** ($5.0 \times 10^{-5}$ M, $\lambda_{ext}$ = 413 nm) in $CH_3OH$ before (black) and after the addition of 2.0 equivalents of anions as tetrabutylammonium salts.*

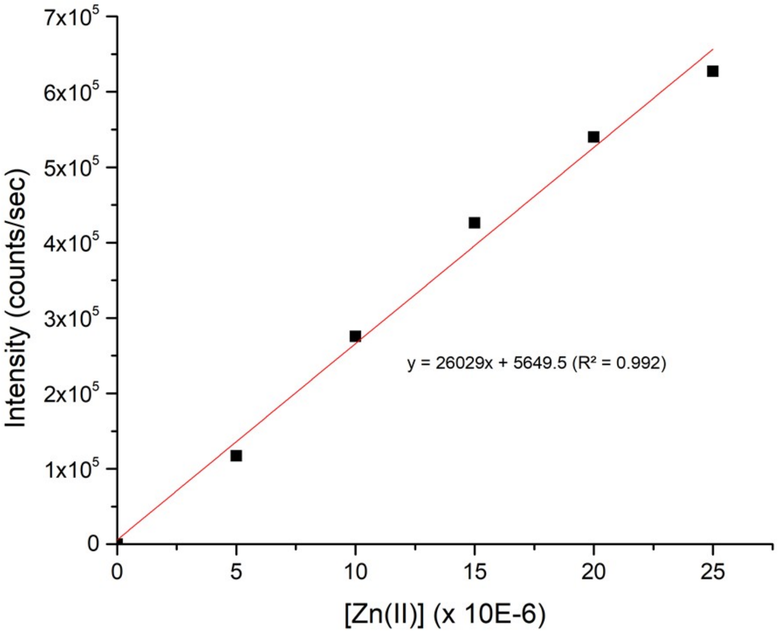


*Figure S13: Normalized response of fluorescence signal of 1 ($5.0\times10^{-5}$ M, $CH_3OH$) in the presence of the increasing amount of Zn(II) (5 – 25 $\times10^{-6}$ M, ACN) ($\lambda_{ex}$ = 413nm; $\lambda_{em}$ = 470nm)*

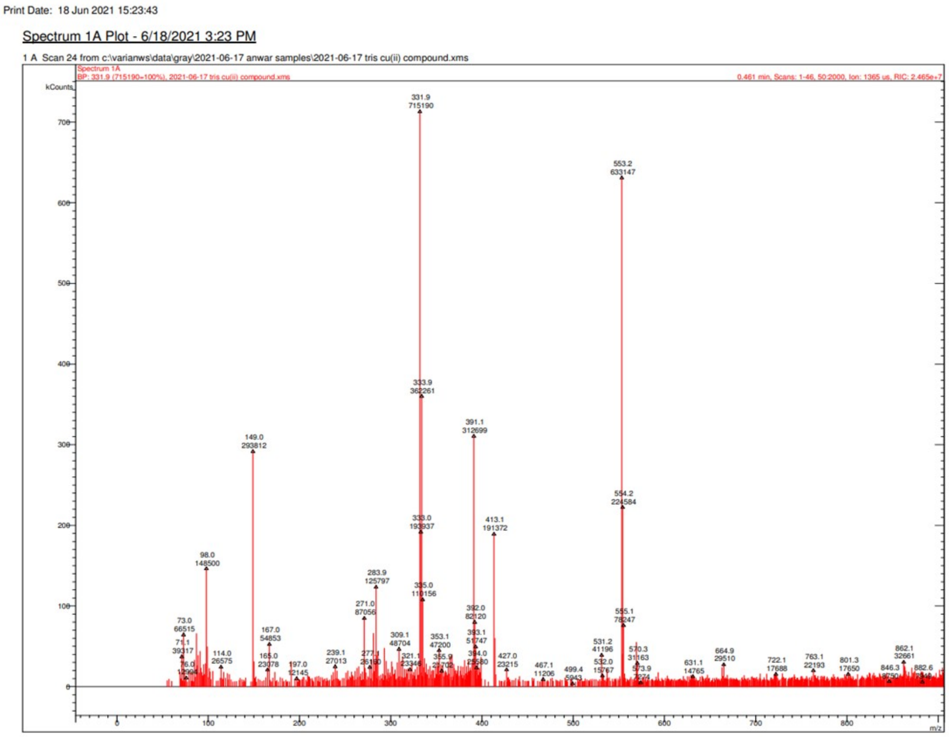


*Figure S 14: Electrospray mass spectrum (ESI-MS positive) of Cu(II) complex of compound **1**.*

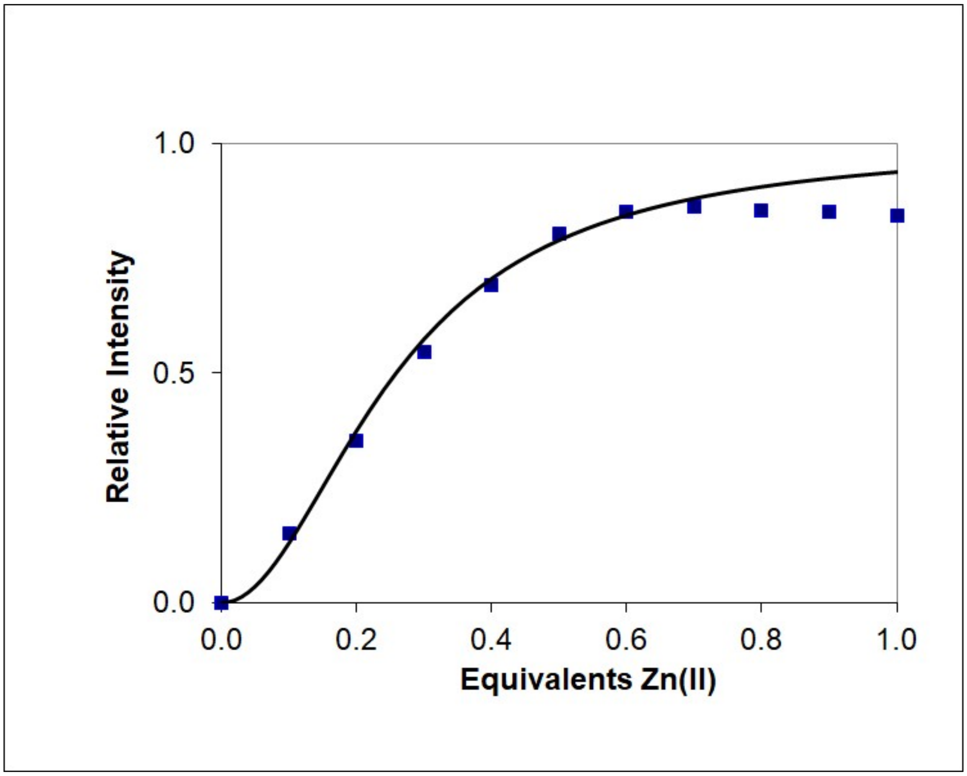


*Figure S15: Relative fluorescence intensity at 470 nm on the added equivalent of Zn(II); best fit for 2:1 compound 1:Zn(II) association. Association constant for 2:1 best fit equal 6.0 x $10^9$ $M^{-2}$ (±10%).*

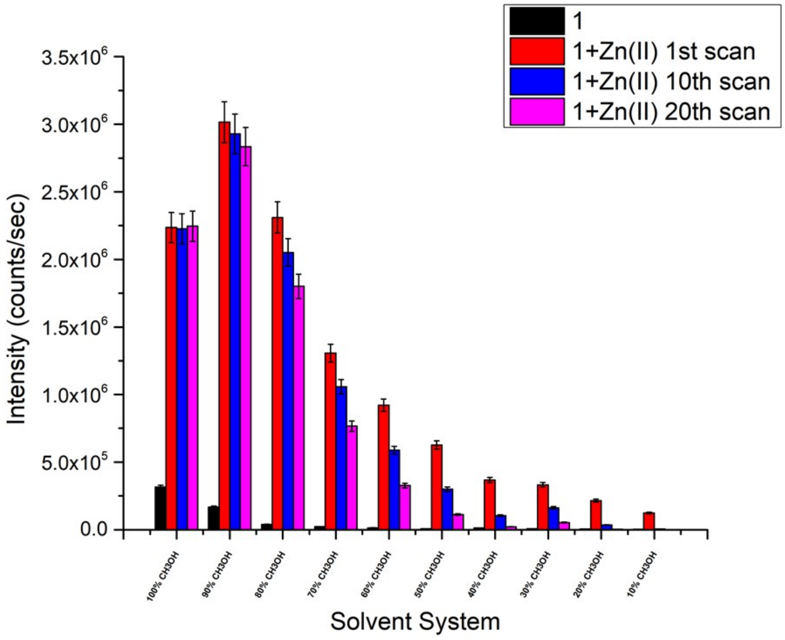


*Figure S16: Relative fluorescence intensity of compound 1 at 470 nm on addition of 2.0 equivalents Zn(II) as perchlorate salts in different fraction of $CH_3OH$ and $H_2O$ (0.1 M HEPES buffer, pH = 7.3) showing the loss of emission at higher content of water. 20 scans with interval of 1 min, $10^{th}$ scan: approx. 15 mins, and $20^{th}$ scan: approx. 30 mins.*

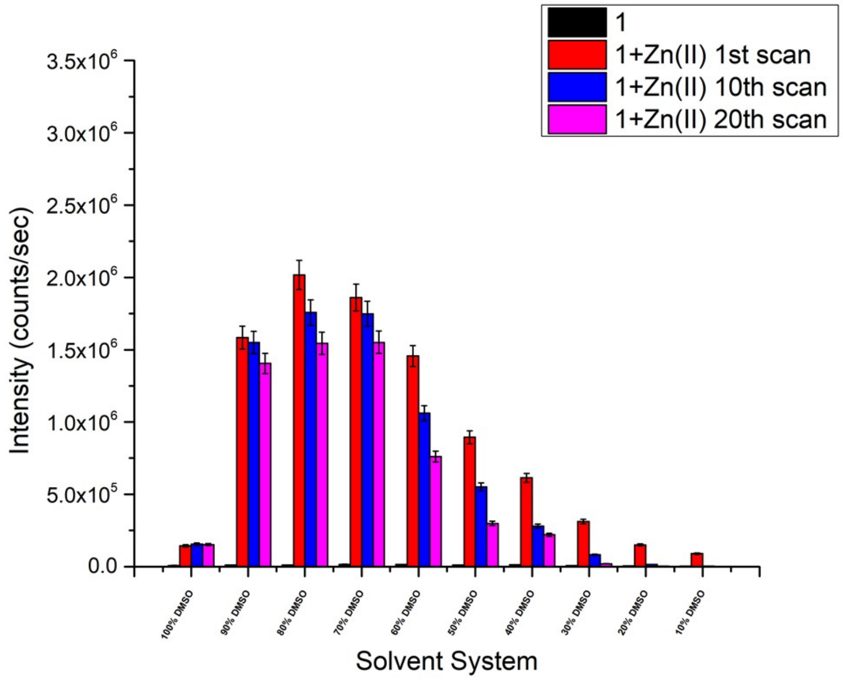


*Figure S17: Relative fluorescence intensity of compound 1 at 470 nm on addition of 2.0 equivalents Zn(II) as perchlorate salts in different fraction of DMSO and $H_2O$ (0.1 M HEPES buffer, pH = 7.3) showing the loss of emission at higher content of water. 20 scans with interval of 1 min, 10$^{th}$ scan: approx. 15 mins, and 20$^{th}$ scan: approx. 30 mins.*

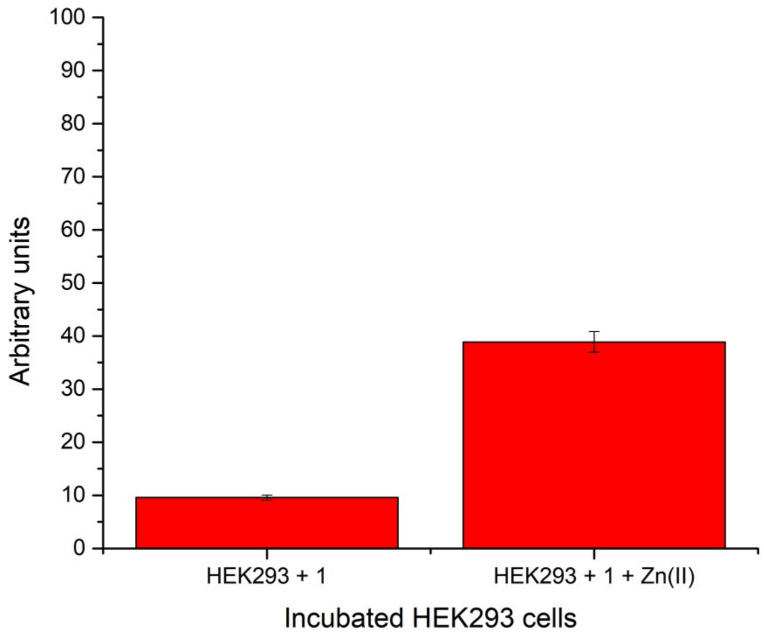


*Figure S18: Mean fluorescence intensity(MFI) of compound 1 in HEK293 cells calculated using Fiji software.*

Job's Plot

5 x $10^{-5}$ M of the compound **1** in methanol and 5 x $10^{-5}$ M Zn(II) as acetate salt in acetonitrile solution were prepared from concentrated stock solutions and 0.25 – 3 mL of **3** and 0 – 2.75 mL of Zn(II) solution were taken in a cuvettes to make total volume of 3 mL and fluorescence spectra were recorded.

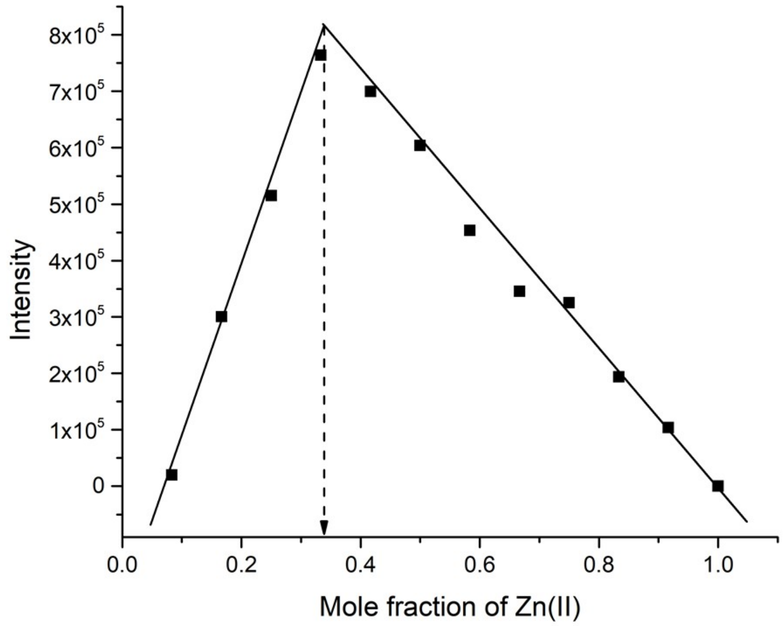


*Figure S19: Job's plot of **1** with Zn(II) with a maxima near to 0.33 confirms the formation of $L_2$Zn(II) complex. Fluorescence intensity monitored at 470 nm vs. a function of the molar ratio of [**Zn(II)**]/([**1**] + [**Zn(II)**]), $\lambda_{ex}$ = 413nm.*

## Calculation of Quantum Yield

$$\phi_{sample} = \phi_{reference} X \frac{A_{sample}}{A_{reference}} X \frac{Abs_{reference}}{Abs_{sample}} X \frac{\eta_{sample}{}^2}{\eta_{reference}{}^2} \qquad (1)$$

Quantum Yield of Compound 1:

$$\phi_1 = 0.27 X \frac{1.74 x 10^6}{6.93 x 10^7} X \frac{0.247}{0.042} X \frac{1.36^2}{1.36^2}$$

$$or, \ \phi_1 = 0.27 X 2.51 x 10^{-2} X 5.88 X 1 = 0.0398 \cong 0.04$$

Quantum Yield of Zinc complex:

$$\phi_1 = 0.27 X \frac{7.86 x 10^7}{6.93 x 10^7} X \frac{0.247}{0.157} X \frac{1.36^2}{1.36^2}$$

$$or, \ \phi_1 = 0.27 X 1.13 X 1.57 X 1 = 0.479 \cong 0.48$$

## Calculation of Detection Limit

$$Detection\ Limit = \frac{3\,\sigma}{K} \qquad (2)$$

$$or,\ Detection\ Limit = \frac{3\ x\ 240.145}{26029}\ X\ 10^{-6} = \ 2.77\ x\ 10^{-8}\ M$$
